# Tracking Plasticity: Effects of Long-Term Rehearsal in Expert Dancers Encoding Music to Movement

**DOI:** 10.1371/journal.pone.0147731

**Published:** 2016-01-29

**Authors:** Rachel J. Bar, Joseph F. X. DeSouza

**Affiliations:** 1 Department of Psychology, York University, Toronto, ON, Canada; 2 Centre for Vision Research, Department of Psychology, Department of Biology, Neuroscience Graduate Diploma Program, Graduate Program in Interdisciplinary Studies, Canadian Action and Perception Network (CAPnet), York University, Toronto, ON, Canada; IIT—Italian Institute of Technology, ITALY

## Abstract

Our knowledge of neural plasticity suggests that neural networks show adaptation to environmental and intrinsic change. In particular, studies investigating the neuroplastic changes associated with learning and practicing motor tasks have shown that practicing such tasks results in an increase in neural activation in several specific brain regions. However, studies comparing experts and non-experts suggest that experts employ less neuronal activation than non-experts when performing a familiar motor task. Here, we aimed to determine the long-term changes in neural networks associated with learning a new dance in professional ballet dancers over 34 weeks. Subjects visualized dance movements to music while undergoing fMRI scanning at four time points over 34-weeks. Results demonstrated that initial learning and performance at seven weeks led to increases in activation in cortical regions during visualization compared to the first week. However, at 34 weeks, the cortical networks showed reduced activation compared to week seven. Specifically, motor learning and performance over the 34 weeks showed the typical inverted-U-shaped function of learning. Further, our result demonstrate that learning of a motor sequence of dance movements to music in the real world can be visualized by expert dancers using fMRI and capture highly significant modeled fits of the brain network variance of BOLD signals from early learning to expert level performance.

## Introduction

Understanding what happens in the brain during the process of learning and practicing a set of skills has important implications for society and health [1–4]. Such knowledge has provided insight into potential tools to assist people who have been affected by brain damage and neurodegeneration [5,6]. Further explorations into the effects of rehearsal may provide greater insight into the neural substrates of learning, a subject relevant to both damaged and healthy brains.

In the area of motor rehearsal specifically, however, much of the research to date appears to either focus on initial acquisition [7] or expert/habit level processes [8,9]. This approach negates the continuous processes occurring over time and results in very different interpretations of what occurs in the brain as a result of rehearsal [7,8,10]. Studies looking at pre- and post-practice states have generally shown that rehearsal leads to increased brain activation in task-related regions [6,11]. In contrast, studies that have compared experts (e.g. a professional dancer or musician) and non-experts suggest that achieving expertise in a motor task results in decreased activation in task-related regions in the brain [12,13]. As well, several studies in practice or habit formation have found shifts in regions of activation over a rehearsal period [14], most often implicating an increased reliance on subcortical regions [15–17]. With respect to motor learning in particular, ambiguity in understanding how plasticity is involved in learning limits the development of effective treatments to rehabilitate the damaged or diseased brain [1,18–20]. Research suggests that understanding the neural underpinnings of complex motor tasks, such as learning a novel dance, is a fruitful model to study motor learning in the real world [21–23]. Therefore, the aim of the present longitudinal study was to examine brain activation patterns for continuous learning effects of long-term rehearsal of complex dance motor sequences over eight months.

As a first step toward this end, we conducted functional magnetic resonance imaging (fMRI) scans to measure Blood-Oxygen-Level-Dependent (BOLD) contrasts in professional ballet dancers while they learned a novel dance to music. The scans were conducted four times over a 34-week rehearsal and performance period. While in the MRI, the dancers listened to the music their choreography was set to and were asked to visualize their dance movements in time with the music. Because prior research has shown that visualization of motor sequences activates similar brain areas as when actually physically executing such movement [24,25], we expected our music-visualization task to activate brain networks involved in learning and performing the dance. We therefore predicted that over the 34-week rehearsal/performance period we would find changes in BOLD signal in areas of the brain associated with initial learning, rehearsing, and performing the dance, including regions involved in audition, motor and habit formation [26,27].

## Materials and Methods

### Participants

Eleven people volunteered for our study. Six professional ballet dancers, including the dance’s choreographer, all with at least two years of professional dance training, were recruited from the National Ballet of Canada’s apprenticeship program (one female, five males, mean age 28.3, range 19–50 years, mean years of dance experience 18.8). However, the one female subject was not able to participate in the end because of a concern that her braces would interfere with functional scanning. Five controls matched for previous dance experience (three females, two males, mean age 30, range 19–48 years, mean years of dance experience 19.6) were also recruited, bringing the total number of included subjects to 10.

The first scan took place after four rehearsals of the dance. The second scan took place a week later, after nine rehearsals. The third scan took place seven weeks after initial acquisition, after the dancers had performed the piece on stage 16 times. The fourth scan took place 34 weeks after initial acquisition. At the time of the fourth scan, the dance had been performed on stage a total of 36 times.

It is important to clarify that two out of the five dancers in the experimental group were unable to attend the first scanning session, and the results for the initial acquisition scan is composed of data from only three subjects. All subsequent scanning sessions in the experimental group included all five dancers.

### Ethics Statement

York University’s ethics committee approved the study (e2013-313), and written informed consent was obtained from all participants in accordance with the committee’s guidelines.

### Equipment and Scanning Procedure

A 3T Siemens Tim Trio MRI scanner was used to acquire functional and anatomical images using a 32 channel head coil. T2*-weighted echo planar imaging using parallel imaging (GRAPPA) with an acceleration factor of 2X with the following parameters: 32 slices, 56 × 70 matrix, 210 mm × 168 mm FOV, 3 × 3 x 4 mm slice thick, TE = 30 ms, flip angle of 90°, volume acquisition time of 2.0 s, was used. There were a total of 240 volumes per scan. Echo-planar images were co-registered with the high-resolution (1 mm^3^) anatomical scan of the subject’s brain taken at the end of each session (spin echo, TR = 1900 ms, TE = 2.52 ms, flip angle = 9°, 256 X 256 matrix). Each subject’s head was restrained with padded cushions to reduce movements.

While in the scanner, participants wore headphones to hear the music: J.S. Bach's *Concerto in C major* [28]. In total, the dance was 6.92 minutes long, but only the final minute of the music was used in our study. Both the music-visualization and motor tasks employed a blocked design with 60 seconds *ON* and 30 seconds *OFF* states. *ON* states were alternated five times. These tasks were analyzed using a Random Effects General Linear Model (RFX GLM) in BrainVoyager QX (Brain Innovation v2.1.1.1542, Maastricht, The Netherlands) with the boxcar function convolved with a double gamma hemodynamic response function. Following statistical analysis of the BOLD signal data was conducted in MATLAB (The MathWorks Inc., version 7.10.0.499, R2010a) and SPSS (Statistical Package for the Social Sciences, IBM SPSS Statistics, version 22).

### Task Procedure

While in the scanner there were two tasks: (1) a music-visualization task cued by music, and (2) a motor localizer task cued by a visual stimulus. During the music-visualization task, the dancers were told to listen to the music and visualize themselves dancing from the internal perspective. The choreographer, who was a retired dancer, was also told to visualize the dance as if he too was dancing it. Unlike the dancers, the controls only had one scan each and were told to visualize themselves dancing when they heard the music. Similar to the control subjects’ scans, during the dancers’ first scanning session, they were not advised about what part of the music they would hear and not told specifically to visualize the steps of their dance. These music-visualization task scans (n = 23) from all subjects (n = 10) were analyzed using a Random Effects General Linear Model (RFX GLM) in BrainVoyager QX with the boxcar function convolved with a double gamma hemodynamic response function.

In the motor localizer task, participants were instructed to move their right toes, extending and contracting them at a rate of 1 Hz, when they saw the word “wiggle” appear on the screen inside the MRI. During all testing, a fixation cross was on the screen. In this motor task, participants needed to keep their eyes open in order to see the visual cue to start moving their toes. However, because the cue was auditory in the music-visualization task, the participants were told they could keep their eyes open or closed if they needed to be able to visualize their dance, and four out of the five dancers reported that they closed their eyes when listening to the music. Each block was repeated five times and thus, both the music-visualization and motor localizer tasks were each a total of 7.5 minutes.

Immediately following the completion of each MRI scan, similar to previously developed visualization tests (e.g. The Vividness of Mental Imagery Questionnaire [29]), we asked participants to subjectively rate their success at visualizing the dance while in the scanner. Although participants did report occasionally losing their place in the dance momentarily, all of them reported they were confident they had visualized the dance while they heard its music during the scan. We also performed a visualization ability test for the dancers after the fourth scanning session.

### Visualization Training and Assessment

To prepare subjects for the visualization task during the scans, we provided the dancers with a 20-minute workshop on visualization the week before beginning the study at their studios. During this workshop the difference between internal and external visualization was explained. The external perspective is visualizing yourself in third-person, as though you are a spectator of the scene [12]. The internal perspective, on the other hand, is a first-person experience of the action being visualized [12]. It has been demonstrated that visualization using the internal perspective may be more similar to actual motor action, with respect to location of activity in the brain [12,30], so it was important that the dancers were coached on how to do this properly. Following the explanation, the dancers were given two examples to practice in the workshop and directed to switch back and forth between the two perspectives, so they could clearly differentiate between them. Following this workshop dancers who volunteered for the study were asked to practice visualizing the dance from the internal perspective and keep track of how often they did this between scans.

To assess the participants’ visualization ability we developed an objective test of each subject’s visualization skill and tested each dancer on the day they participated in scan four. Immediately after exiting the MRI scanner each dancer was played their music and at randomly pre-selected pauses was asked to either verbally explain or demonstrate the step they were doing at the moment the music was paused. Their answers were compared to a video of the dancers performing the piece. Of the 25 visualization trials given to each participant (five set pauses, repeated five times, making a total of 125 possible correct trials) there were only three trials in which one dancer reported he was not sure where in the dance the music had been paused. All 122 other trials were accurate within a two second range. These results suggest the dancers were able to accurately visualize their dance movements while completing the music-visualization task in the scanner at time point four.

### Preprocessing

Functional data were superimposed on anatomical brain images, aligned on the anterior commissure-posterior commissure line, and transformed into Talairach space in BrainVoyager QX. Functional data from each run were screened for motion or magnet artifacts to detect eventual abrupt movements of the head. In addition, we ensured that no obvious motion artifacts (rims of functional activation) were present in the activation maps from individual participants. We also filmed the participants while in the scanner so we had record of whether there were any noticeable physical movements while being scanned. Only one noticeable foot movement from one participant across the scans was found during scan 1 in the music-visualization task, but every functional scan was motion corrected to correct for possible head movements that participants may have made.

### Statistical Analysis

Participants’ functional data were analyzed within a multi-subject random effects general linear model (RFX GLM). The RFX GLM compared the activity of the music-visualization task and fixation blocks for all 10 subjects and thus used 23 scans to provide an unbiased sample of our functional visualization maps. The RFX GLM is designed explicitly to examine the variability of a condition across subjects [31]. It is usually executed in multiple steps, where the first step of the analysis collapses multiple scans of the same subject, thus resulting in a mean estimate for that subject per condition [31]. This is done so that results may be generalized to the population. We next computed the RFX GLM of the music-visualization task across every subject and a second RFX GLM of the motor localizer across every subject. In the final step we computed a region of overlap from the music-visualization and motor localizer tasks within the Supplementary Motor Area (SMA), showing that this overlapped cortical region was activated by both tasks. All music-visualization regions were computed at the false discovery rate (FDR) corrected for multiple comparisons (*p* < 0.05). Similarly, our auditory region and subcortical regions were created from the same scans (at *p* < 0.05, FDR corrected). The BOLD GLM activation between task and fixating blocks appeared as activation maps superimposed onto an anatomical image of a participant’s brain. Within each brain region, voxel activity was extracted for all time points (initial acquisition, weeks 1, 7, and 34). However, the first scan (initial acquisition) was not included in our repeated-measures ANOVAs due to the fact that two dancers missed this scan. Thus, our longitudinal examination of learning compared weeks 1, 7, and 34 using repeated-measures ANOVAs. Linear and quadratic statistical fits analyses and post-hoc *t*-tests with Bonferroni corrections of alpha levels for multiple comparisons were also conducted.

## Results

**Fig 1:**
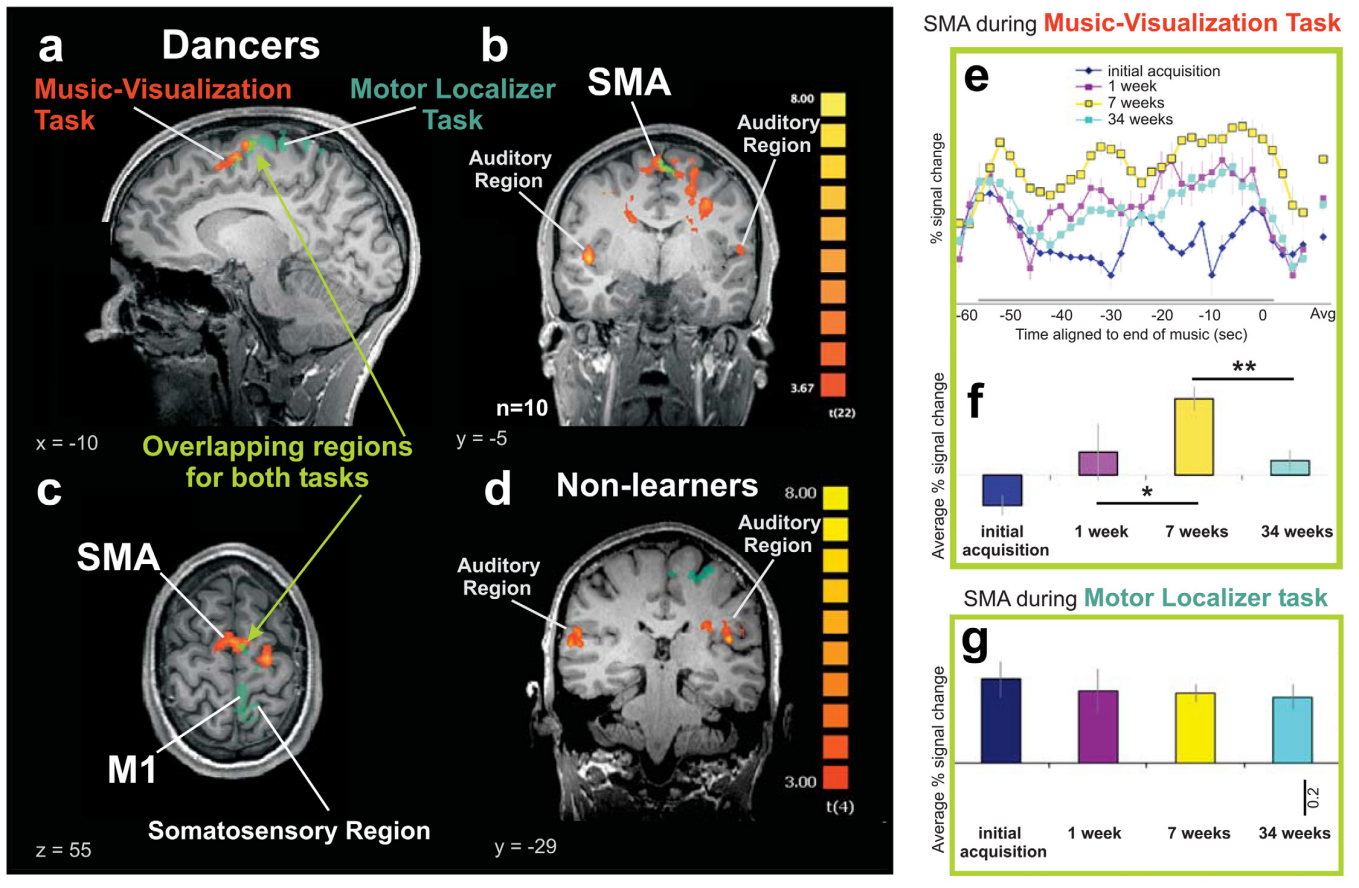
Supplementary motor area (SMA) in music-visualization and motor tasks. All figures show the average activity across participants. **a-c,** The overlapping region of interest (light green) was created with the GLM contrasts (music-visualization task > baseline) in orange and (motor task > baseline) in green, using a random effects general linear model (RFX GLM) from the third scan as described in the methods, *p* < 0.05, FDR corrected. The brain images have been made with a cluster threshold of 88 voxels (k>88). **d**, Activity in our five control subjects matched for dance experience in the same two tasks as described in the methods above. No overlapping SMA region was found between the tasks in any slices–a representative slice shown here. **e,** Music-visualization task: percent of BOLD signal change extracted from the SMA (overlapping region) of all dancers at *p* < 0.05, FDR corrected. **f**, Music-visualization task: Average BOLD signals of each of the four scans in SMA region (taken from the gray period highlighted at the bottom of 1e and the averaged points in 1e). Signal increased from second to third scan (*p* < 0.05, paired *t*-test) and decreased from third to fourth scan (*p* < 0.05, paired *t*-test). **g**, Motor task: Average BOLD signal of the four scans in same SMA voxels. No significant changes were found across the scans in this task. All error bars represent the standard error of the mean (s.e.m.). Scale bar represents 0.2 percentage BOLD signal change. * signifies *p* < 0.07 trend and ** signifies *p* < 0.05 corrected for Bonferroni multiple comparisons.

We first conducted GLMs for the two tasks described above and then examined a region that overlapped between the two tasks within the supplementary motor area (see green voxels in SMA region shown in Fig 1A). The SMA is a brain region located in the superior frontal gyrus, involved in planning, sequencing and executing motor sequences [32–34]. Thus, a region of analysis was made from this functionally defined overlapping region as it provided an independent localizer for our tasks. A repeated measures ANOVA in this region revealed that BOLD signals in week 1, week 7 and week 34 were significantly different from each other, *F*(2, 8) = 6.157, *p* < 0.05, η^2^ = 0.61. Posthoc *t*-tests revealed that during the music-visualization task, there was a trend towards an increase in activation from the week 1 to the week 7 (*p* = 0.07, Bonferroni corrected for multiple comparisons at an alpha significance level at *p* < (0.05 / 3) = *pCorr* < 0.017). Most importantly, there was a significant decrease of activation from week 7 to week 34 (*p* = 0.006, when comparing the yellow and turquoise curves/bars in Fig 1E and 1F). In contrast, as the controls were matched for dance experience but were not learning the choreography and were only asked to visualize dance to this music at the first time point, no overlapping activation was found between the two tasks in SMA (Fig 1D).

**Fig 2:**
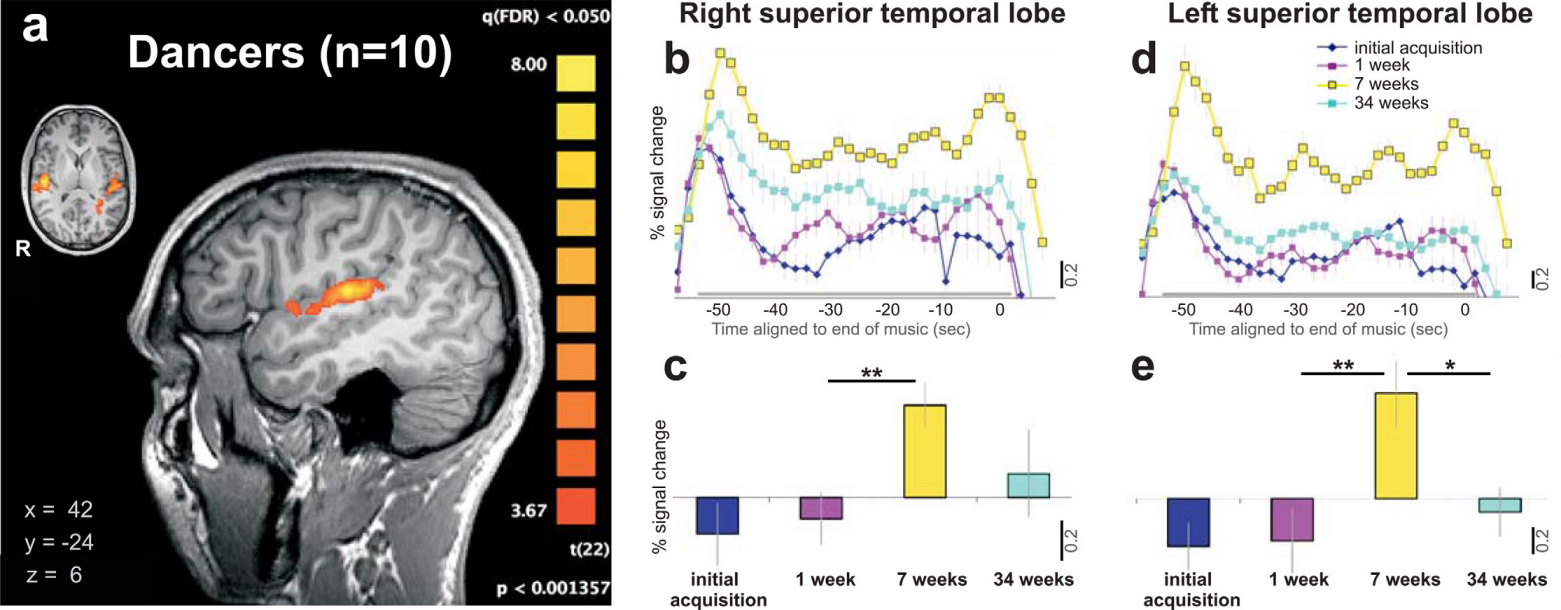
Auditory activation in music-visualization task. All figures show the average activity across participants using an RFX GLM, *p <* 0.05, FDR corrected. **a**, Auditory activation in the music-visualization task (parasagittal view is of right hemisphere). **b**, Music-visualization task: Percent of BOLD signal change across the four scans in right superior temporal lobe. **c**, Music-visualization task: Average BOLD signals of each of the four scans in right superior temporal lobe (taken from the gray period highlighted at the bottom and averaged in 2b). Signal increased from the second to the third scan (*p* < 0.01, paired *t*-test). **d**, Music-visualization task: Percent of BOLD signal change across the four scans in left superior temporal lobe. **e**, Music-visualization task: Average BOLD signals of each of the four scans in left superior temporal lobe (taken from the gray period highlighted at the bottom of 2d and the averaged points in 2d). Signal increased from the second to the third scan (*p* < 0.05, paired *t*-test), and decreased from third to fourth scan (*p* < 0.05, paired *t*-test). All error bars represent the s.e.m. Scale bar represents 0.2 percentage BOLD signal change. * signifies *p* < 0.025 trend and ** signifies *p* < 0.05 corrected for Bonferroni multiple comparisons.

Auditory activation in the superior temporal lobes of both hemispheres was also found in all participants during the music-visualization task (Fig 2A, Fig 1B and 1D). A repeated measures ANOVA in these regions also revealed main effects of time in each hemisphere, *F*(2, 8) = 7.252, *p* < 0.025, η^2^ = 0.64 (Right), *F*(2, 8) = 7.882, *p* < 0.025, η^2^ = 0.66 (Left). Further posthoc comparisons revealed significant increases in these regions between the week 1 and week 7 scanning sessions in each hemisphere (*p* = 0.006; *p* = 0.013; with *pCorr threshold* < 0.017). There was a decreasing trend for the BOLD signals from the week 7 to week 34 scanning session in the left hemisphere (*p* = 0.024, which was approaching significance at the Bonferroni correction of *pCorr threshold* < 0.017, Fig 2D and 2E).

**Fig 3:**
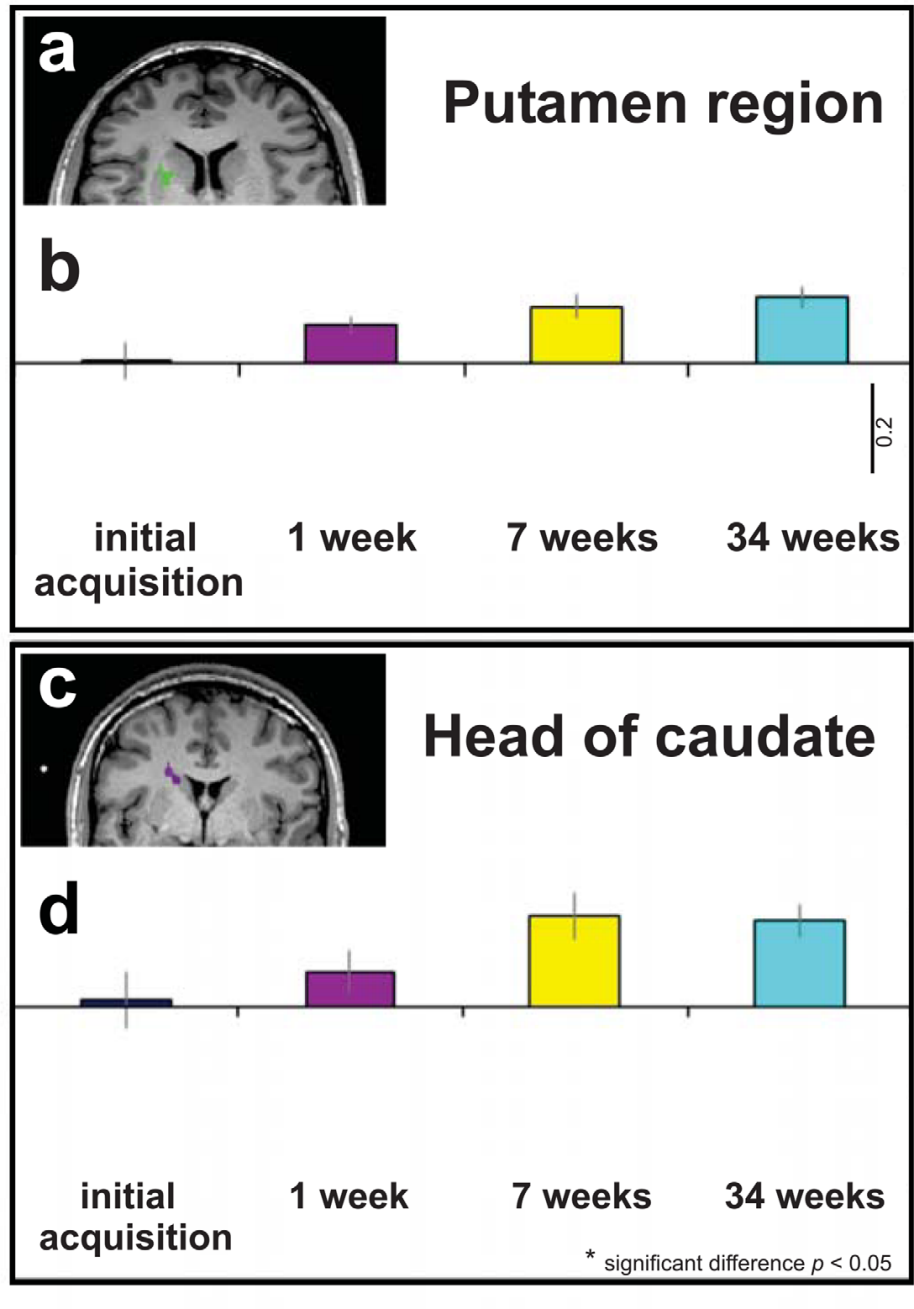
Subcortical activation in music-visualization task. All figures show the average activity across participants using the contrast music-visualization task > baseline with the RFX GLM, *p <* 0.05, FDR. The brain images have been made with a cluster threshold of 20 voxels (k>20). **a,** Music-visualization task: Right putamen region (x = 21, y = 10, z = 15). **b**, Music-visualization task: Average BOLD signals of each of the four scans in the region identified as right putamen. No significant changes were found in this region across scans. **c**, Music-visualization task: Caudate region (x = 18, y = -4, z = 26). **d**, Average BOLD signals of each of the four scans in the region identified as right caudate. All error bars represent the s.e.m. Scale bar represents 0.2 percentage BOLD signal change.

Subcortical basal ganglia regions, including the putamen (Fig 3A) and caudate (Fig 3C) were also found to be activated during the music-visualization task at the same thresholds as the cortical regions described above (FDR < 0.05). A repeated measures ANOVA in these regions revealed that unlike activation in SMA and auditory regions, which showed decreases at week 34, there were no significant decreasing trends in these subcortical regions from week 7 to week 34, Caudate *F*(2, 8) = 3.426, *p* = 0.138, η^2^ = 0.461; Putamen *F*(2, 8) = 1.624, *p* = 0.256, η^2^ = 0.289 (Fig 3B and 3D, when comparing yellow and turquoise bars, N.S. as in the previous cortical areas). Thus, although our basal ganglia regions (caudate/putamen) were activated for the music-visualization task at the FDR <0.05 threshold and a reduced cluster size (k>20), there was no significant modulation at any time of the three time points examined.

**Fig 4:**
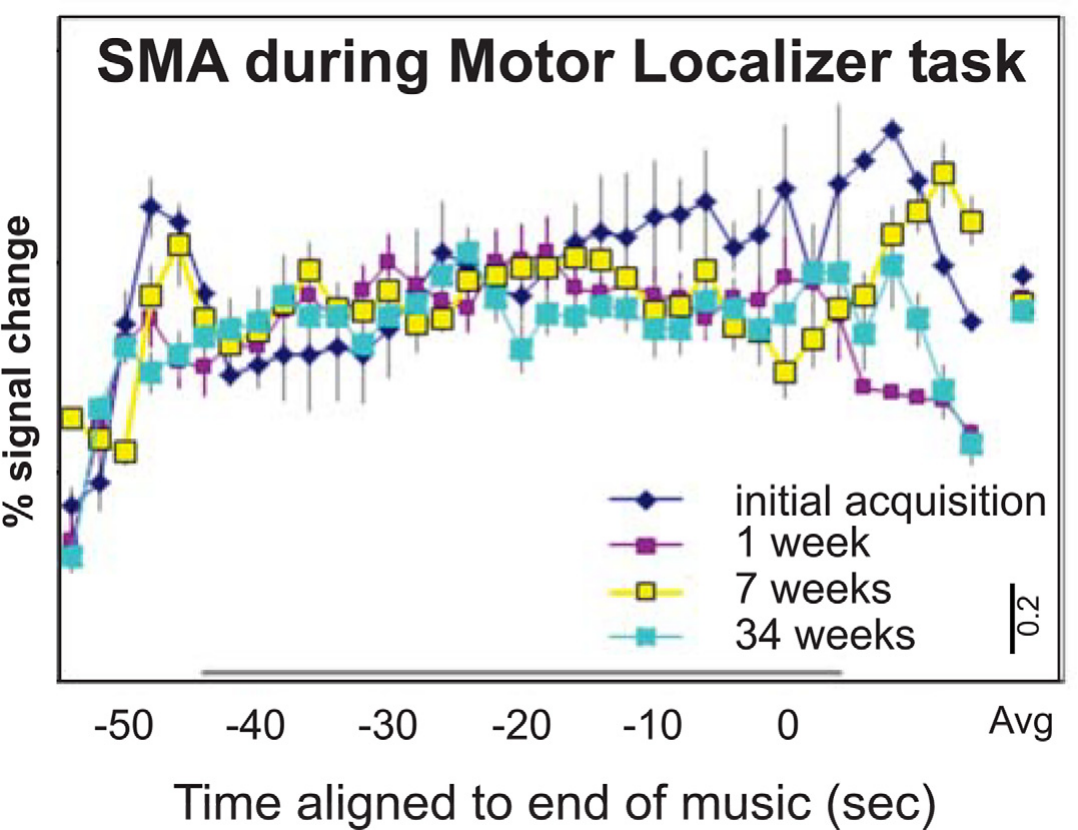
SMA activation in motor task. All values were extracted using an RFX GLM, *p* < 0.05, FDR corrected. Motor task: Percent of BOLD signal change across the four scans. All error bars represent the s.e.m. All conventions the same as Fig 1E.

Analysis comparing the same SMA region across the time points in the motor localizer task revealed no significant changes in activity across the three time points [*F*(2, 8) = 0.886, *p* = 0.498, η^2^ = 0.37; Figs 1G and 4]. As the motor task was not practiced outside the scanner in between the four scanning sessions across 34 weeks (see time course in Fig 4), these findings further support the notion that the changes in BOLD signal found in the music-visualization task (Figs 1F, 2C and 2E) may be related to the increase in rehearsal and performance experience of the dancers as measured in our music-visualization task and not merely a consequence of the task being repeated four times in the MRI scanner.

Additionally, at the functional map thresholds above, the dorsal premotor cortex (PM) in the left hemisphere (Fig 1C) also showed significant activation but did not show any significant modulation with learning the dance across time (*F*(2, 8) = 1.423, *p* = 0.296, η^2^ = 0.26). Since previous research within motor learning has shown that the motor cortex was also involved in motor skills learning [4] we used the localization of voxels within our motor localization task maps in (Fig 1A, 1B and 1C–dark green/blue region) and then extracted the signal from the music-visualization scans. This analysis showed that there was no significant effect of motor learning across our last three time points in our study from the music-visualization in dancers from the motor localization of the right foot (*F*(2, 8) = 2.284, *p* = 0.164, η^2^ = 0.36).

Our subjects learned choreography and performed their dance a maximum of thirty-six times over 34 weeks. Thus we next examined whether the pattern of the BOLD brain signals across the time period was best fit with a linear and/or quadratic function to the data across time from all functionally mapped brain regions (FDR<0.05). The BOLD signals for the SMA brain region (Fig 1F) showed a highly significant quadratic fit to the group means with over 94 percent of the variance accounted for described by the size effects, *F*(1,4) = 63.590, *p* = 0.001, η^2^ = 0.941 and no linear trend *F*(1,4) = 0.114, *p* = 0.753, η^2^ = 0.028 (see Table 1). There were also significant quadratic fits for the other cortical regions during the music-visualization task, motor regions localized by the foot, left PMd and both auditory regions (Fig 2C and 2E) with no significant linear trends for all these regions. The basal ganglia regions did not have a significant quadratic fit in either functionally localized region (Fig 3, Table 1) and the linear fits to the group means approached a trend (caudate, *F*(1,4) = 3.426, *p* = 0.138, η^2^ = 0.461; putamen, *F*(1,4) = 3.204, *p* = 0.148, η^2^ = 0.410).

**Table 1:** Quadratic fits from the time points of 1, 7 and 34 weeks of BOLD signals across regions.

Region	MS	F stat	Sig	Partial Eta Squared
SMA	0.354	63.6	0.001	0.941
Motor region from localizer	0.052	16.2	0.016	0.802
AUD right	0.877	14.6	0.019	0.785
PMd left	0.105	9.2	0.039	0.697
AUD left	3.132	8.5	0.043	0.681
CAUDATE right	0.015	1.6	0.272	0.288
PUTAMEN right	0.009	0.8	0.410	0.175

SMA = Supplementary Motor Area, motor region (localized from the foot motor task), PMd = Dorsal premotor cortex, AUD = auditory region.

## Discussion

These preliminary findings suggest that the timing of learning real world sensorimotor tasks can be tracked from cortical and subcortical regions using fMRI in experts performing a novel dance sequence to music. More specifically, our findings suggest that rehearsal of a complex motor sequence initially leads to increasing activity within a network of SMA and auditory cortical regions up to at least the seven-week point, and then decreasing activation between the 7th and 34th week. This does not necessarily mean that the region is no longer significantly involved. Neurons within these regions may have become more efficient, changed their connection weights, or chunked together for efficiency [35–43]. In any case, there appear to have been significant changes in the way the dance was being visualized across learning and performance, and to our knowledge, this is the first time that the transition from initial stages of learning to expert level has been followed over eight months in a real world setting [1,44].

Our results also suggest that habit formation, or increased familiarity with a motor sequence, as measured using a music-visualization task activates putamen and caudate regions of the basal ganglia, but only in the right hemisphere. No changes in signal across learning within these subcortical regions of the basal ganglia were found. One possible explanation of this result could be the known involvement of subcortical regions, such as the basal ganglia, in habit formation [14–17,39]. As the dance became extremely well-learned and thus more of a habit for the dancers (due to sustained, repetitive, and intense rehearsal), SMA and left auditory cortical regions became less involved; while subcortical regions potentially maintained the habit. Alternatively, lack of changes across time in these regions may be due to the small amount of voxel activations in these regions which may have underpowered the analyses. Future work in this area is therefore needed to uncover the processes occurring at the subcortical levels, but is beyond the scope of this preliminary report.

### Further consideration of SMA findings

Previous research suggests the SMA region fires before a movement sequence is initiated [30–32,45], is implicated in the performance of complex motor sequencing [46], is involved in motor preparation, and is also activated through motor imagery [47]. More recently, SMA neural activations that were computed using dynamic motor sequences to assist brain machine interfaces for reaching demonstrated a neural signature that is quantitatively and qualitatively very different than PM/M1 [48–50]. In line with these previous findings, we found significant activation of this region in both the music-visualization and motor tasks. However, unlike the motor task where minimal learning was taking place between scans across eight-months, we found significant changes in activation across time points for the music-visualization task. Our findings suggests that SMA may play a key role in the learning of new motor sequences cued by auditory signals associated with music, but that memory traces of these dance movements, once their familiarity is experienced more automatically or habitually through continued performance, is likely represented elsewhere in the brain’s circuitry, possibly with more links to subcortical regions. This may be similar to the analogy used by dancers called “muscle memory” once a performance is overlearned.

### Further consideration of subcortical findings

Evidence has suggested that the basal ganglia may play a direct role in the development of habit formation [51]. Early studies with animal models found that lesions to the caudate nucleus lead to delayed and altered behaviors [52–54]. Other studies have demonstrated that in people with Parkinson’s disease (PD), where basal ganglia function has been compromised, habit learning occurs through structures associated with declarative memory (mainly the prefrontal cortex) as opposed to healthy controls that demonstrated greater caudate activation during habit formation [55]. While significant cortical network activity decreased as the dance became more familiar to the dancers in this study, subcortical basal ganglia activity did not vary across our experiment. Additionally, only the right hemisphere was activated for the basal ganglia during learning and performance of their dance as measured during visualization. One notable observation is a right hemisphere lateralization of encoding of motor habits over the 34 weeks. This is observed first in the right auditory cortex peaking at seven weeks but without significant drop in signal by 34 weeks, whereas the left hemisphere auditory cortex showed a trend towards a decrease from the 7^th^ to the 34^th^ week. This is visible when comparing the yellow waveforms for the 7^th^ week with the turquoise waveforms from the 34^th^ week in Fig 2B and 2D.

This trend of having a right hemisphere showing increased signal also shows up in the subcortical regions. This right hemisphere lateralization flows with two other research studies examining expertise in dancers and opera singers–thus coding music to movement–showing lateralization to the right hemisphere in cortical [56] and right subcortical basal ganglia regions [57]. Since our task in essence was the learning that was done in a real world dance, which included travelling through the space of the studio and theatre during learning and performance, it is likely this has to do with external spatial layout of the visualized dance through space [22,58] or the efference copy/feedback of reward expectancy for the visualized dance back to auditory cortex from the salience network regions [59–63].

### Further consideration of auditory findings

Weis and colleagues found that auditory cortex receives feedback signals when the task was well-learned possibly from anterior insula nodes within the saliency network [59,63]. In particular, one of their studies [63] showed the right auditory cortex to be more lateralized which may correspond to whether the outcome of the visualization of the dance confirmed the dancer’s expectations within our data. In the current study, the lateralization within the right hemisphere is tentative. Thus future research will be needed to tease out these more subtle effects of expertise related to timing of motor movements to music [64].

Cortical auditory activation found in our data is also consistent with previous findings. Platel and colleagues [65] discovered preferential activation in the left hemisphere during a music identification task. Specifically, the left inferior frontal gyrus and superior temporal gyrus were activated when the music was well known to listeners. Not only did we find a similar location in the left auditory cortices of our dancers, but we also found a significant increase of activation in the superior temporal lobe from week one to week seven and trend toward a decrease from week seven to week 34. These results may therefore suggest that music familiarity, similar to movement familiarity, may initially be developed by cortical networks, but at some point, memory of music either requires less energy and/or is maintained elsewhere in the brain.

### Limitations

Our results must be considered as preliminary evidence given the limitations of this longitudinal, within-subject study. The sample of four dancers and a choreographer is a small experimental group, even for an imaging study. Although the choreographer did not perform the dance, this study explores the long-term effects of a combination of physical and mental rehearsal. Given that the choreographer actively participated in every rehearsal, we believe the results from this particular participant reflect the overarching effects of rehearsing a movement sequence to music. As well, we scanned the dancers and choreographer 18 times, adding to the power of our longitudinal analysis. With our five control subjects, we had the same number of subjects scanned as Brown and colleagues’ seminal study of the neural basis of dance [7]. Furthermore, the patterns of changes reported were found in each participant’s functional activity, suggesting that we had enough power to demonstrate the results within each subject and across 34 weeks.

The controls in this study, although matched for dance experience, did not learn a dance across time. While the dancers that learned a dance piece were scanned at four time points our dance-experienced controls were tested just once. This means there was no control for repeated-scanning effects shown across time. In an attempt to address this, we demonstrate that in the motor localizer task, no significant change across the four scans was found (see Fig 4). Even though this appears to address some of the control variables in question, it does not control for any music-visualization learning effects. Previous research has suggested that visualization of movement activates similar regions and networks of the brain as when physically engaged in the same task [24,27,30]. However, our study is not able to determine whether the changes in the dancers’ brain activity were the results of physical rehearsals of their dance or the visualization rehearsals of their dance. Future replication of this study should therefore follow age and experience matched controls across the same length of time as the dancers. This would allow for any differences in the neural networks and patterns of activation to be more clearly attributable to the learning and performance of the novel choreography and not potentially confounded by some other unknown variable.

A possible limitation of this study is that we may have been underpowered. In our current study we had 10 participants with a total of 23 imaging sessions across all dancers. This may have not been enough power to show significant activation in the whole visuomotor cortical and subcortical networks described in prior studies [34–35,66] involving expert dance performance [8,9], (see [1,44,67] for recent reviews). But we do show very significant activations and a compelling pattern of signal changes. Thus, while our findings focus on three main regions of the brain, by no means do we claim that no other brain regions/networks are involved in coding motor learning of a complex dance.

Additionally, our study, like Brown and colleagues’ [7], had no visual stimulus present while the dancers visualized or did their dance since all of our participants only listened to the music while visualizing/imagining their dance. In fact, four of five ballet dancers closed their eyes to visualize or imagine their dance in the scanner, so they had no visual stimulus at all. This may aid us to explain why there was less activation along the frontal-parietal circuitry [66] than has typically been observed in prior studies, as this network is thought to be primarily driven by vision [66]. A direct comparison between visualizing/imagining dance from music alone and with the addition of a visual cue (e.g. recorded dance) would therefore further clarify the distinctions between these methodological approaches.

Additionally, the only neuroimaging studies showing professional dancers doing their own choreography involved only four subjects moving in the lab while simultaneously recording EEG and body movements [68] and a case study where only one professional dancer did improvised visualization of dance to music that he selected as extremely familiar and then compared to two age matched controls [69]. In contrast, the professional dancers in our study did perform their learned dance on stage to a live audience, and at three times over eight months they visualized their dance within the MRI. Therefore our subject pool is comparable or greater to any published neuroimaging study examining expert dancers performing to an audience [68].

Thus, although there were many potential shortcomings to our preliminary report, we do show a very telling and significant longitudinal story of a realistic dance learning paradigm that was being learnt in the natural world. We show an evident profile of learning through the cortical dance network as our professional dancers learned and performed their choreography over eight months. The modeled quadratic fits to the brain imaging data show an inverted “U-shaped” learning profile with most the variance being explained first by SMA, than motor cortex, right auditory cortex, left dorsal premotor cortex and finally left auditory cortex regions (shown in Table 1). This learning profile gives us insight into the relative importance of the neural networks involved in visualizing the learned/performed dance.

### Cortical regions involved in learning dance

Our data-driven longitudinal approach showed SMA to have the highest correlation to learning/visualization of real dance choreography with music across time. In contrast, left dorsal premotor is suggested as the most critical brain region for motor learning (reviewed by Hardwick and colleagues [27]). As well, a more recent study showed that 4 days of dance training in dance novices also showed similar cortical regions within the AON (action observation network) and bilateral superior temporal sulci activation. The post-training correlated to ratings of liking the dance [70] that they experienced and maybe related to the feedback signals returning to auditory cortex discussed earlier [63]. Thus, our study now refines the dance visualization network for motor learning of performed choreography suggesting that SMA is the major player across time [64,71] with premotor, motor regions and auditory regions all significantly involved. Additionally, the motor learning paradigm employed in our study was part of the dancers’ job description and rehearsal was conducted in a natural setting (i.e. the ballet studio and on stage) rather than in a laboratory setting. A further strength of this paradigm therefore is its ecological validity [44].

## Conclusion

Previous research has shown that rehearsal leads to an increase of activation in the short-term and a decrease of activation when task expertise is achieved. However, to our knowledge, the transition from the initial stages of learning to expert level has not yet been followed long term. The present study therefore helps clarify this transition by demonstrating that even among experts, rehearsal of a dance motor sequence will lead to an increase of activation for several weeks, before an eventual decline of activation in significant cortical regions. Although limitations of this study warrant caution when interpreting our preliminary longitudinal results, given the significant learning related changes found, replication of this study with a larger sample will likely help to further elucidate networks associated with motor learning and longitudinal memory for sequences of movements coded to music.
